# The Status of Genetic Resources and Olive Breeding in Tunisia

**DOI:** 10.3390/plants11131759

**Published:** 2022-07-01

**Authors:** Olfa Saddoud Debbabi, Fathi Ben Amar, Sameh Mnasri Rahmani, Francesca Taranto, Cinzia Montemurro, Monica Marilena Miazzi

**Affiliations:** 1 Banque Nationale de Gènes, Boulevard du Leader Yesser Arafet, Charguia 1, Tunis 1080, Tunisia; mnasrisameh@yahoo.fr; 2 Laboratory de Production Oléicole Integrée (POI-16-IO-03), Olive Tree Institute, University of Sfax, Aéroport Road, Sfax 3000, Tunisia; 3 Laboratory of Improvement and Protection of Olive Genetic Resources, Olive Tree Institute, University of Sfax, Aeroport Road, Sfax 3000, Tunisia; fathibenamar@gmail.com; 4 Institute of Biosciences and Bioresources (CNR-IBBR), Via Celso Ulpiani 5, 70126 Bari, Italy; francesca.taranto@ibbr.cnr.it; 5 Department of Soil, Plant and Food Sciences (DISPA), University of Bari, Via Amendola 165/A, 70126 Bari, Italy; cinzia.montemurro@uniba.it

**Keywords:** *Olea europaea* L., molecular markers, olive oil quality, biotic and abiotic stresses, genetic resources

## Abstract

The olive tree, an iconic symbol of the Mediterranean basin, is the object of growing international interest in the production of olive oil for the world food market. In Tunisia, which is the fourth-largest producer of olive oil in the world, the production of olives and olive oil is of great socio-economic importance. Cultivation is widespread from north to south, but it is carried out using traditional techniques that results in extremely irregular production levels. To maintain their competitiveness on the international market, Tunisian producers must improve the quality of the oil through breeding plans that enhance the rich genetic heritage that is still not adequately exploited. The objective of this review is to present the state of olive breeding in Tunisia, illustrating the opportunities available for a better use of the rich Tunisian genetic heritage, the challenges it must face, and the need to multiply the efforts for sustainability, even in the light of the challenges posed by climate changes.

## 1. Introduction

Olive (*Olea europaea* L. sub. *europaea* var. *europaea*) is one of the main crop and fruit tree in the Mediterranean region, where wild (the ancestor), feral and domesticated forms are widespread [1]. Olive is the pillar of the Mediterranean agro-ecosystems, given its great economic, social and cultural importance [2,3]. The crop is cultivated on more than 12 million hectares worldwide [4], displaying a wide variability witnessed by more than 2600 different varieties for oil and/or table fruits, which are conserved at the World Olive Germplasm Bank [5].

**Figure 1:**
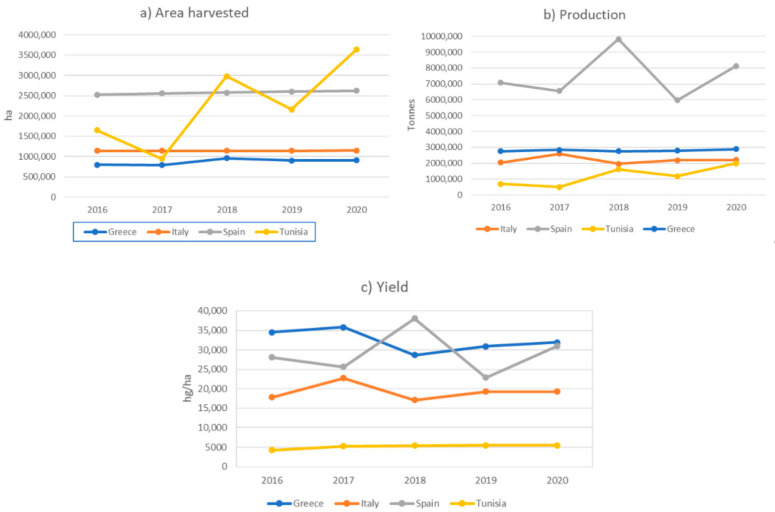
Graphic representation of olive production statistics for the world’s three largest producers and for Tunisia (FAOSTAT 2020). Tunisia is the fourth-largest olive-oil-producing country in the Mediterranean Basin, with 82 million olive trees covering an area of 1.84 million hectares. Olive oil represents 40% of the overall value of agronomic exports of the country.

Olive is considered the most suitable and best-adapted crop to the Mediterranean environment, where it encompasses 80% of the worldwide olive tree area. Spain is the world’s largest producer of olive oil with more than 35% of global production, followed by Greece, Italy, and Tunisia (Figure 1). These four countries, together with Portugal, are also the largest exporters of olive oil [6].

However, the production quantity and quality of the olives in the Mediterranean basin are strongly influenced by climate conditions [7]. In recent years, numerous projections have indicated the Mediterranean basin as a “hotspot” of climate change due to warming and drying trends that are affecting olive production [8,9]. The adaptation of varieties changes according to their genetic background but also to the area of cultivation, and strongly depends on the pedoclimatic conditions prevailing in each olive grove.

As part of the climate change adaptation plan, genetic resources are expected to play a significant role in mitigating impacts and supporting efforts to achieve food security and nutrition goals. Knowledge of the olive’s evolutionary history and the role of its wild forms in domestication represents a basic prerequisite for the improvement of modern varieties through the introduction of favorable alleles from wild oleaster or other subspecies of *O. europaea* [10,11]. Therefore, the recovery of genetic diversity and the identification of genetic regions associated with important agronomic traits linked to phenology, resistance to stress and diseases, yield and quality of the oil, become fundamental. In recent decades, more than 100 olive collections have been realized around the world, while 23 olive germplasm banks created by the International Olive Council, house over 1700 well-identified and characterized varieties [12]. These ex situ preserved resources offer an inestimable wealth of plant material and have already contributed enormously to the development of future olive breeding programs in several Mediterranean countries [13,14,15,16]. In the meantime, advances in genomics and phenomics are accelerating the exploitation of genetic resources through the study of loci associated with important traits in several crops [17,18,19,20] including olive [21,22,23,24], also to perform product traceability [25,26,27].

The challenge of olive breeding must consider several aspects that are interconnected. The need for modern highly productive and mechanized agriculture has become more pressing in recent decades, leading to an increase in demand for olive varieties more suitable for the transition from extensive to intensive olive plantations [28]. The main request of olive growers is to have plants that guarantee high productivity and quality, but also suitable for modern cultivation systems. Thus, they have to be characterized by a reduced vegetative vigor to favor mechanical operations, a reduced alternate bearing, and an increased self-fertility rate [29]. Breeding for fruit traits focuses on set, drop, size, pit size, yield per tree, and the flesh-to-pit ratio [30,31], while breeding for oil quality aims to increase oleic acid, phenols, and α-tocopherol content [32,33].

A major challenge has also arisen in relation to the growing environmental impact of climate changes associated with abiotic stresses such as cold, salinity, and drought, as well as new or resurgent pests and diseases such as *Verticillium dahliae*, *Mycocentrospora cladosporioides*, *Bactrocera oleae*, *Prays oleae*, and *Pseudomonas syringae pv. savastanoi* [34,35,36]. A new emergency for world olive growing emerged in 2013 in southern Italy, where the new disease, the Olive Quick Decline Syndrome (OQDS), was caused by the bacterium *Xylella fastidiosa* subsp. *pauca* (*Xf*), and seems unstoppable due to the current unavailability of suitable treatments, making the discovery of new sources of resistance in olive trees a feasible strategy [37].

Olive breeding is complicated, long, and laborious due to several limiting factors that affect it negatively. The olive tree has a long juvenile phase that can last up to 15–20 years in natural conditions [38]. It is highly heterozygous, with a high number of chromosomes (n = 23), and a 70% repetitive component of DNA, consisting of 30% tandem repeat sequences and 40% transposable elements [39]. Furthermore, according to the observations on the progenies obtained in cross-breeding programs, most of the components for olive oil quality show extreme variability [40,41]. However, olive-breeding programs have been implemented with success around the world, resulting in the release of several new commercial varieties, such as ‘Kadesh’, ‘Barnea’, ‘Maalot’, ‘Askal’ in Israel [30], ‘Chiquitita’ in Spain [42], ‘Arno’, ‘Tevere’ and ‘Basento’ in Italy [43]. In this country, a cultivar selected in the 1970s as clonal rootstock from seedlings of cv. Frantoio, the FS17, has been now identified as tolerant of OQDS and it is seen as an opportunity for olive cultivation in OQDS-infected areas [37].

## 2. Importance of Olive Tree in Tunisia

Tunisia is one of the world’s top olive-oil-producing countries, occupying the second place after Spain in terms of the area dedicated to olive plantations, with 1.8 million hectares and 86 million olive trees [44]. More than 75% of its olive oil production is export-oriented [45], primarily to the European Union, and the policies are aimed at further strengthening the competitiveness of this product in the international market. With this goal, the country has become the fourth-largest producer of organic olive oil (25,000 t in 2009) with an organic olive grove covering 48,000 ha in particular in the Sfax region, which represents 22% of the total national production of organic olive oil [46]. In addition, it has recently obtained a “Protected designation of origin (PDO)” quality label for Teboursouk olive oil (3 March 2020, records of the World Intellectual Property Organization (WIPO)). Nevertheless, the proportion of olive oil packaged for export remains low, and Tunisia hopes to double oil exports in the next 5 years by applying the Hazard Analysis and Critical Control Point (HACCP) management system [47].

In this paper, we aim at exploring the state of olive breeding in Tunisia, illustrating the opportunities available for better use of the rich Tunisian genetic heritage, and analyzing the needs and possible responses to the challenges posed by modern olive culture.

## 3. Diversity of Olive Genetic Resources in Tunisia as Reservoir for Varieties Selection

Over the centuries, due to its strategic geographical position in the Mediterranean Sea, Tunisia has become a reservoir of genetic diversity of the olive tree. The different civilizations that have followed one another, in fact, have favored the exchange and flow of genetic resources from various foreign countries [48,49].

**Figure 2:**
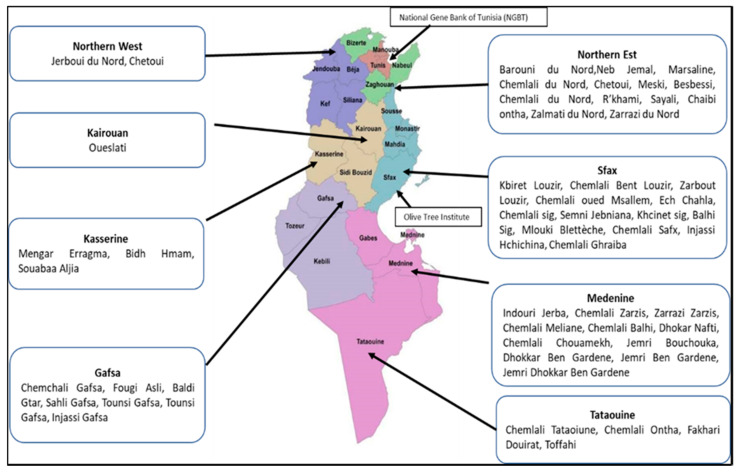
Map of Tunisia with the distribution of the most important olive tree varieties. The main olive varieties cultivated in the north of the country are Chetoui, Sayali, and Gerboui, while cv. Chemlali and Oueslati are typical of the central-southern areas, where also the cv. Zalmati, Zarrazi and Tounsi are largely grown. The map also indicates the location of Tunisian olive germplasm is represented by over 200 varieties of olives that are conserved at the National Gene Bank of Tunisia (NGBT) (Tunisi) and the Boughrara Collection implemented at Olive Tree Institute in Sfax.

**Figure 3:**
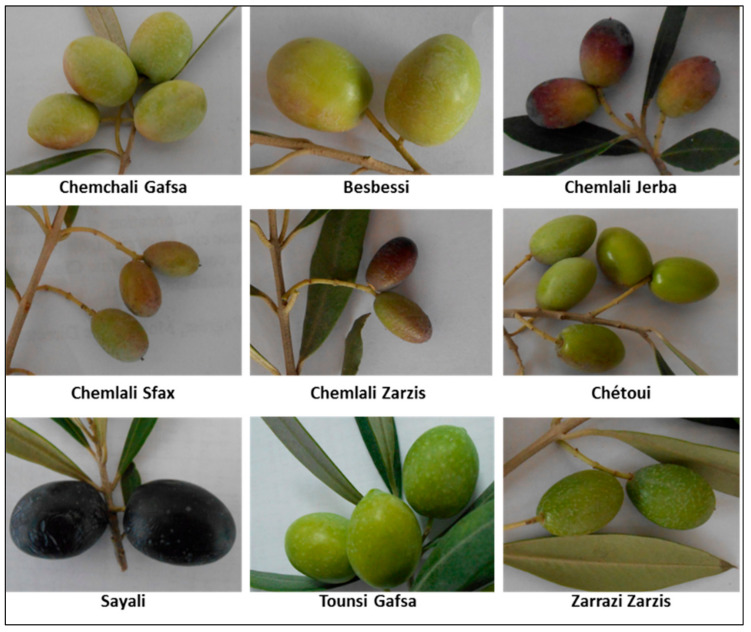
Some of the most common olive varieties cultivated in Tunisia.

Nowadays, the Tunisian olive germplasm is represented by over 200 varieties of olives that are conserved at the National Gene Bank of Tunisia (NGBT) and at Boughrara Collection implemented at Olive Tree Institute in Sfax (Figure 2 and Figure 3). This collection includes the most important oil varieties, such as Chemlali, Chetoui, Chemchali, etc., and 10 table varieties. This product, which has great importance in the Tunisian diet, is characterized by greater size, taste, and organoleptic properties that make them suitable for table consumption. They are represented by the cultivars Barouni, Besbessi, Meski, Tounsi, Marsaline, Injassi Gafsa, Souabaa Aljia, Beldi Zaghouan, Picholine, and Zarrazi, all native to the Northern areas of the country [50].

## 4. Identification and Authentication

The characterization and authentication of cultivars is the first step toward their valorization. Tunisian olive germplasm characterization has been for a long time based on morphological traits. These, despite being strongly influenced by environmental factors, have allowed the most important Tunisian varieties to be described and cataloged [51,52]. Alongside the morphological characterization, an accurate biochemical characterization of the main monovarietal oils has also been performed for the most important Tunisian varieties [53,54]. Today, molecular markers have become the tool of choice for crop fingerprinting, genetic diversity, and population structure studies, and to set up efficient tools for their product’s traceability and authenticity [55,56]. In Tunisia, most of the molecular studies have been carried out by using AFLP [57], ISSR [58], and mostly SSRs [59,60,61,62], while very few are based on SNP markers [63,64] (Table 1).

## 5. Olive Breeding in Tunisia 

In Tunisia, olive breeding has aimed, so far, at satisfying the demand from local farmers and markets; today, it is required to meet the needs of the global economy, competitiveness, labor availability, climate changes, and emerging diseases. In recent years, Tunisia has faced growing problems with wilting and plant dieback, mainly caused by *Fusarium oxysporum*, *F. solani*, *V. dahlia,* and other fungi [77,78,79,80]. This issue has spurred the implementation of programs of selection to control diseases and reduce crop losses [68]. In 2021, as part of a screening for genotypes potentially resistant to *X. fastidiosa*, a study on genetic diversity in a large collection of Mediterranean olive trees highlighted a strong genetic relationship of a Tunisian cultivar with the Italian varieties ‘Leccino’ and ‘FS17’, which are resistant to *X. fastidiosa*, thus indicating their potential, not only for Tunisian but for the entire Mediterranean basin’s olive breeding [81].

Even the wild olive is considered a valuable reservoir of alleles favorable for breeding to control abiotic and biotic stresses [82,83,84]. Wild olive forests are native to Tunisia and wild/feral olive forms can be found in natural and agrarian ecosystems [65]. This germplasm appears promising for resistance to defoliating *Verticillium* [85] and root-knot nematodes [86], as well as drought tolerance [87]. In addition, it is considered a resource for improving olive oil’s quality. Hannachi et al. [69] showed wild olive oils are richer in fatty acids, polyphenols compounds, chlorophylls, and sterols compared to the two most cultivated Tunisian varieties CHEMLALI and CHETOUI.

In the frame of climate changes hitting Mediterranean countries, resistance to drought is considered a crucial trait to be improved in Tunisia, in particular for the aridest regions of the south, where severe droughts occur in summer with increasing frequency [88]. From 1993 to 1996, with the financial support of the International Olive Council, an extensive program of controlled crossings using conventional breeding techniques was undertaken [89]. The program focused on the two main oil varieties, CHEMLALI SFAX and CHETOUI, and the main table variety, MESKI [90]. These varieties are widely grown in rainfed areas forming the Tunisian olive grove landscape. CHEMLALI SFAX is renowned for its productivity and adaptation to different environments, and it is classified as salt tolerant [74]. It has a great ability to exclude toxic ions and to control the net import of salt in shoots; however, it has a poor acid oil composition, containing little oleic acid and a lot of palmitic acid. CHETOUI is mostly cultivated in the North of Tunisia, and it is noted for its very fruity oil characterized by green almond aromas and richness in phenolic compounds [74]. The breeding program of CHEMLALI SFAX was aimed to select new genotypes which maintained the good agronomic qualities of the variety while adding a better acid composition [70]. The crossing program resulted in more than 1500 offspring, which have been evaluated since 1997 for their oleic acid content, and since 2005 for the agronomic value and quality profile of the oil. The program made it possible to select several promising candidates [66,91], proving the superiority of offspring from CHEMLALI SFAX × CV. SIGOISE oils which had profiles in fatty acids and triacylglycerols more favorable compared to CHEMLALI SFAX × CV. MESKI, confirming the high contribution of the cultivar factor to the quality of oil [67,71].

**Figure 4:**
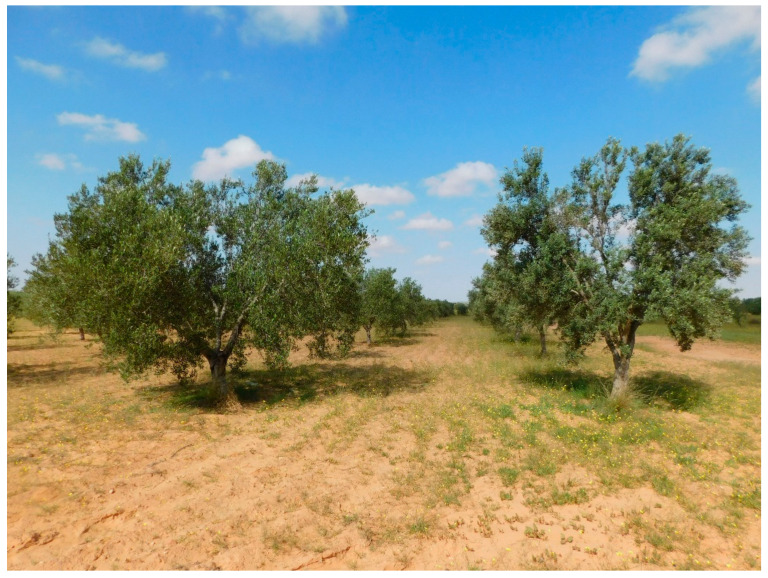
Example of in situ conservation of olive trees in the Tunisian National olive collection of Boughrara, Sfax.

**Figure 5:**
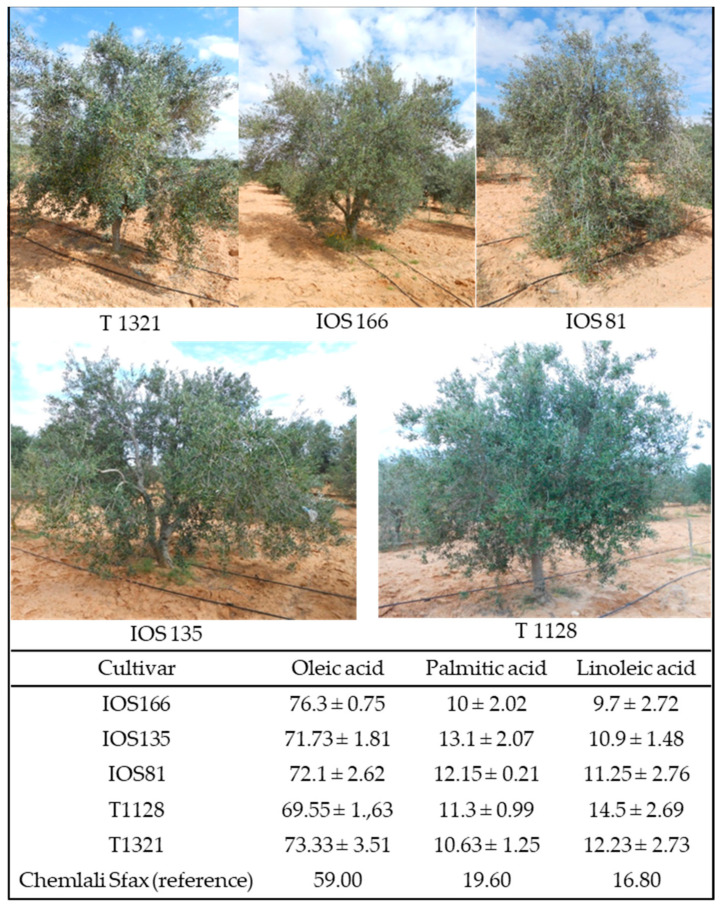
Photos of the five released hybrids (Fathi Ben Amar, Olive Institute, Tunisia) in the frame of a cross-program between cv. CHEMCHALI SFAX with CHEMLALI GAFSA and cv. LUCQUES. In the table, the average performances for the main fatty acids of the released hybrids are indicated.

CHEMLALI SFAX has also been used in a more recent cross-breeding program with cv. CHEMCHALI GAFSA and cv. LUCQUES, as well as by self-pollination, obtaining 11 promising hybrids that have been evaluated starting from 2017, rainfed and under the arid conditions of the Sfax region. This program has resulted in the release of five new cultivars now registered in the Official Journal of the Republic of Tunisia [92] (Figure 4). Compared to reference cultivar Chemlali Sfax, they are characterized by higher productivity, lower content of palmitic acid, and higher content of oleic acid2 (Figure 5) [71]. The evaluation of these hybrids is still ongoing for other agronomic traits with the aim of expanding the number of new varieties available to turn the Tunisian olive sector towards a high-quality level.

Besides the studies on the main cultivars CHEMLALI SFAX and CHETOUI, the investigation of minor and rare Tunisian olive cultivars has also been carried out, as a strategy to enrich and diversify Tunisia’s olive oil production. In 2021, Omri et al. [72] proved the high potential of several minor varieties for quality improvement of oils, showing that these varieties had high content in tocopherol and oleic acid (BAROUNI and CHEMLALI CHOUAMEKH), chlorophyll (FOUGI GTAR and ZARRAZI GTAR), luteolin (ZARRAZI ZARZIS and ZARRAZI GTAR), apigenin (ADHEFFOU), rutin and verbascoside (BOUDAOUD). Similar results were obtained by Chtourou et al. [73] on minor varieties from the area of Sidi Bouzid (SEHLI and CHEMCHALI) and Gafsa (BALDI, BESBESSI, TOUNSI, NEB JMEL and CHEMCHALI), confirming the high potential of Tunisian germplasm for improving Tunisian olive oils.

## 6. Perspectives

Modern olive growing is facing new challenges such as offering healthy products, adapting to climate change, protecting natural resources, and conserving the landscape. Breeding can play a decisive role in achieving these goals, offering new varieties better adapted to biotic and abiotic stresses, and producing high-quality oils.

Today, plant breeding has gone from being a completely phenotype-based process to a genotype-based process [93,94,95]. The information generated using SNP-based approaches may allow us to discover genes/alleles useful for enhancing target traits, while new advanced biotechnologies, such as transformation-based editing methods, allow us to transfer them to elite cultivars. Improvements in transformation and regeneration protocols in a recalcitrant species such as the olive are continually reported, and important traits such as resistance to fungi, flowering, or lipid composition, have already been successfully manipulated [96,97,98].

In Tunisia, olive breeding has partially satisfied the farmers’ requests, obtaining varieties showing olive oil characteristics better than the traditional varieties, but further investigations are needed. By drawing on the rich genetic heritage of the olive germplasm still present in Tunisia, and by using breeding strategies based on advanced genomic approaches, it will be possible to identify genes involved in agronomic, productive and developmental traits (e.g., juvenility, self-incompatibility, ovary abortion, chill response), as well as in the resistance to biotic and abiotic stress. Only by continuing this work there will be further positive effects on the quality of the olive oil in the next decade, with positive impacts also on Tunisian oil exports.

## Figures and Tables

**Table 1:** List of the morphological and genetic studies performed on Tunisian olive.

Type of Characterization		Reference
Morphologic/Agronomic		Trigui and Msallem 2002 [50]
		Laaribi et al., 2017 [51]
		Khabou et al., 2006 [52]

		Hannachi et al., 2010 [65]
		Mezghani et al., 2019 [66]
		Ben Amar et al., 2015 [67]
Biochemical		Rjiba et al., 2009 [40]
		Ben Abdallah et al., 2018 [46]
		Rejeb et al., 2019 [47]
		Grati-Kamoun et al., 2001 [53]
		Zarrouk et al., 2009 [54]
		Guellaoui, et al., 2021 [68]
		Hannachi et al., 2013 [69]
		Ben Amar 2021 [70]
		Dabbou et al., 2010 [71]
		Omri et al., 2020 [72]

		Chtourou et al., 2021 [73]








Molecular	RAPD	Laaribi et al., 2017 [51]
	AFLP	Grati-Kamoun et al., 2006 [57]
		Saddoud Debbabi et al., 2021 [49]
		Laaribi et al., 2017 [51]
		Ben Ali et al., 2011 [58]
		Rekik et al., 2008 [59]
	ISSR/SSR	Fendri et al., 2010 [60]
		Ben Mohamed et al., 2017 [61]
		Abdelhamid et al., 2017 [62]


		Hannachi, et al., 2010 [65]


		Dridi, et al., 2018 [74]

	SNP	Ben Ayed et al., 2014 [63] Ben Ayed et al., 2019 [64]

In two recent papers, Saddoud et al. [75,76] using SSR markers, highlighted a high genetic diversity in Tunisian marginal germplasm, such as that collected in areas of Ras Jbal, Azmour and the oasis of Degache, in the southwest part of Tunisia. Interestingly, authors identified gene pools which are not present in the commercial varieties, suggesting the availability of rich yet unexplored genetic resources that might be related to traits of adaptation to harsh conditions and thus very useful for improving olive resilience to abiotic stresses.

## Data Availability

Not applicable.
